# Surgical Duodenotomy Following Untreated Bouveret Syndrome

**DOI:** 10.7759/cureus.4866

**Published:** 2019-06-10

**Authors:** Adel Hanandeh, Shyam Allamaneni, Alex Shikhman

**Affiliations:** 1 General Surgery, Columbia University School of Physicians and Surgeons at Harlem Hospital Center, Harlem, USA; 2 Surgery, Jewish Hospital, Cincinnati, USA; 3 General Surgery, Jewish Hospital, Cincinnati, USA

**Keywords:** duodenotomy, bouveret syndrome

## Abstract

Bouveret syndrome is caused by the impaction of a gallstone into the duodenum through a cholecystoduodenal fistula. This is typically followed by pyloric obstruction via retrograde migration of the stone, as opposed to anterograde migration, which can result in gallstone ileus. Bouveret syndrome usually presents with nausea, vomiting, and abdominal pain. Pneumobilia is seen on radiographic imaging. Herein, we describe a case of Bouveret syndrome where the diagnosis and treatment were delayed due to the initial patient desire for surgical intervention. Ultimately, duodenotomy was performed after several failed attempts of endoscopic stone extraction.

## Introduction

Bouveret syndrome is a rare complication of chronic cholelithiasis and is defined as gastric outlet obstruction resulting from gallstone impaction in the pylorus following retrograde migration from the duodenum [1]. Alternatively, anterograde migration of the stone results in gallstone ileus. The gallstone finds its way to the duodenum by eroding the wall of the gallbladder and nearby structures, resulting in a cholecystoenteric fistula. Cholecystoenteric fistulas include cholecystoduodenal fistula (most common about 60%), cholecystogastric, cholecystocolic, and choledochoduodenal fistulae. Both gallstone ileus and Bouveret syndrome are very rare complications of bowel obstruction, accounting for only 1% - 4% and 1% - 3%, respectively [1-2]. Although both conditions can occur at any age, they are more common in the elderly due to untreated chronic cholelithiasis. Typical clinical features include nausea and vomiting, abdominal pain, hematemesis, weight loss, and anorexia [1-5]. However, Bouveret syndrome can be missed due to minimal or non-specific symptoms [2]. Diagnosis is usually achieved using abdominal computed tomography (CT), upper endoscopy, or (in some rare cases) surgical exploration [3]. The treatment of Bouveret syndrome requires extraction of the identified obstructing stone via endoscopy or, more commonly, via surgery. Endoscopic therapy can be achieved via mechanical, electrohydraulic, or laser lithotripsy.

## Case presentation

An 88-year-old woman with a history of dementia, end-stage renal disease on hemodialysis, hypertension, and chronic cholelithiasis presented with nausea and non-bloody emesis for two days. No abdominal pain, fever, chills, dysuria, hematuria, melena, or hematochezia were exhibited.

**Figure 1:**
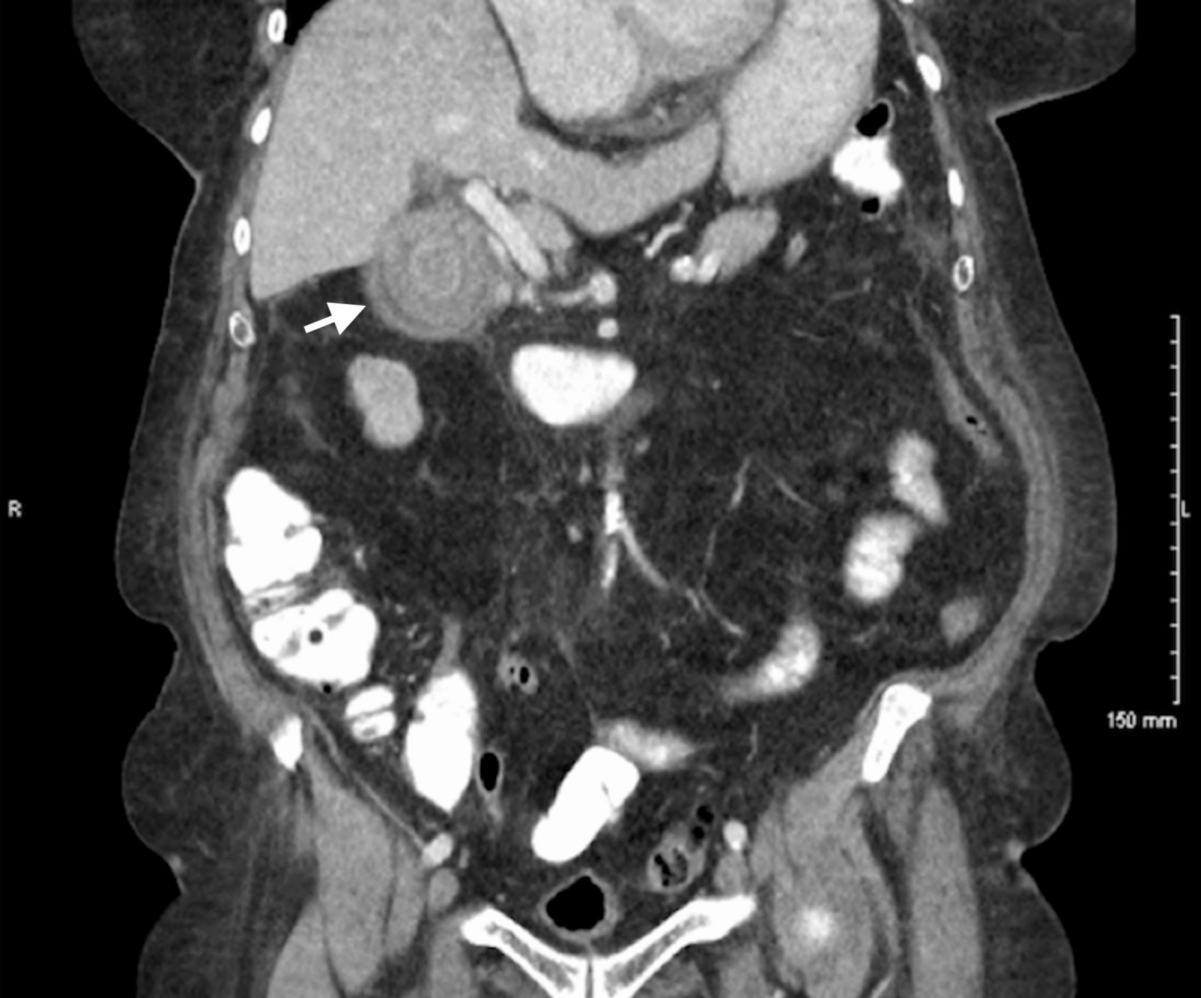
Initial CT scan illustrating the gallstone within the gallbladder (white arrow)

**Figure 2:**
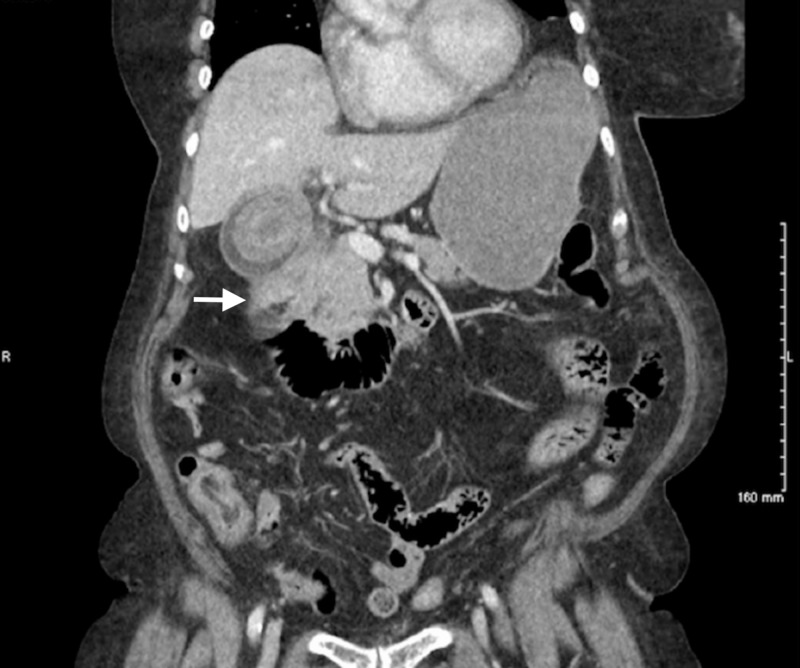
Computed tomography scan obtained one year after initial presentation showing extensive duodenal inflammation (white arrow).

She was hospitalized three years prior for a similar presentation. At that time, a CT scan was obtained which showed a large stone within the gallbladder (Figure 1). During that presentation, the patient refused any surgical intervention and was treated conservatively. One year later, the patient returned with a similar presentation in which a second CT was obtained (Figure 2) which showed a gall stone, as well as inflammatory changes and possible duodenal stricture. Thereafter, an esophagogastroduodenoscopy was performed and showed duodenopathy with a post-bulbar duodenal stricture. It was thought that the cholelithiasis may have caused inflammatory changes of the duodenum and led to the stricture formation. Pathological examination failed to demonstrate malignancy or Helicobacter pylori infection. She was treated conservatively per her request to avoid surgical intervention.

Examination on this admission revealed a core temperature of 98.6, a heart rate of 78, a respiratory rate of 16, and an elevated blood pressure of 171/89. The patient had a normal mental status, respiratory, and cardiovascular examination. Abdominal examination was significant for moderate abdominal distention but otherwise unremarkable. Murphy’s sign was negative. There was no guarding or rigidity, no hernia was observed, and no masses or enlarged liver or spleen were palpated. Laboratory investigations were remarkable for an alkaline phosphatase of 283 unit/L (normal: 25 - 100), serum glucose of 171 mg/dl, and leukocytosis of 16.7 with a left shift. In addition, the renal panel was abnormal with a blood urea nitrogen of 33 and creatinine of 6.3. Other hepatic, hematologic, and metabolic studies were unremarkable.

**Figure 3:**
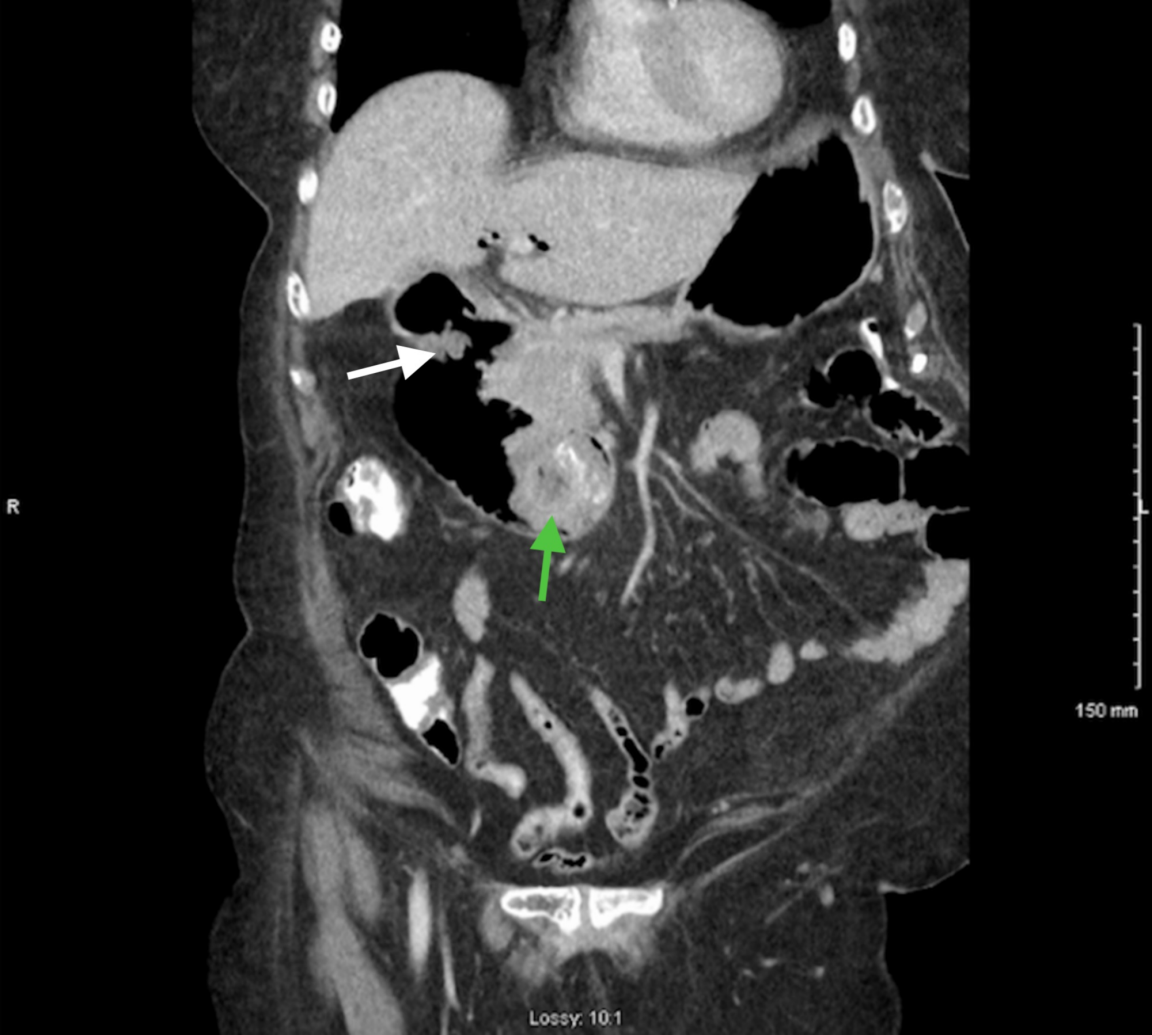
Follow-up computed tomography scan three years after initial presentation showing complete erosion of the stone into the duodenum with gastric distention (white arrow indicating the fistula and green arrow indicating the stone)

An abdominal roentgenogram showed an abnormal gas pattern with distended gastric outline and gastrectasis which was increased compared to her prior studies obtained two years ago. A moderate stool burden was noted throughout the inferior colon and rectum representing fecal impaction. No free air or significant calcification were noted. An abdominal CT scan (Figure 3) showed a large 4 cm gallstone in the duodenal bulb with interval development of air within the biliary tree and common duct. A nasogastric tube was placed and drained > 1 liter of dark, red-tinged fluid. Endoscopic studies revealed a normal esophagus, gastric antrum, and pylorus. Debris and fluid were found in the stomach and were suctioned using a gastroscope. A normal duodenal bulb with a large gallstone (4 - 5 cm) at the apex into the duodenal sweep was found. This was surrounded by mucosal edema. Because of the stone angulation, it was difficult to obtain purchase on the stone. Different devices were used in attempts to free the stone from the duodenum and manipulating it into the stomach. Efforts failed due to the calcified nature of the stone, the angulation of the duodenum, extensive inflammatory changes surrounding the stone, and the size of the stone. Subsequently, surgical management was discussed with the patient and her family who agreed to proceed with the operation.

**Figure 4:**
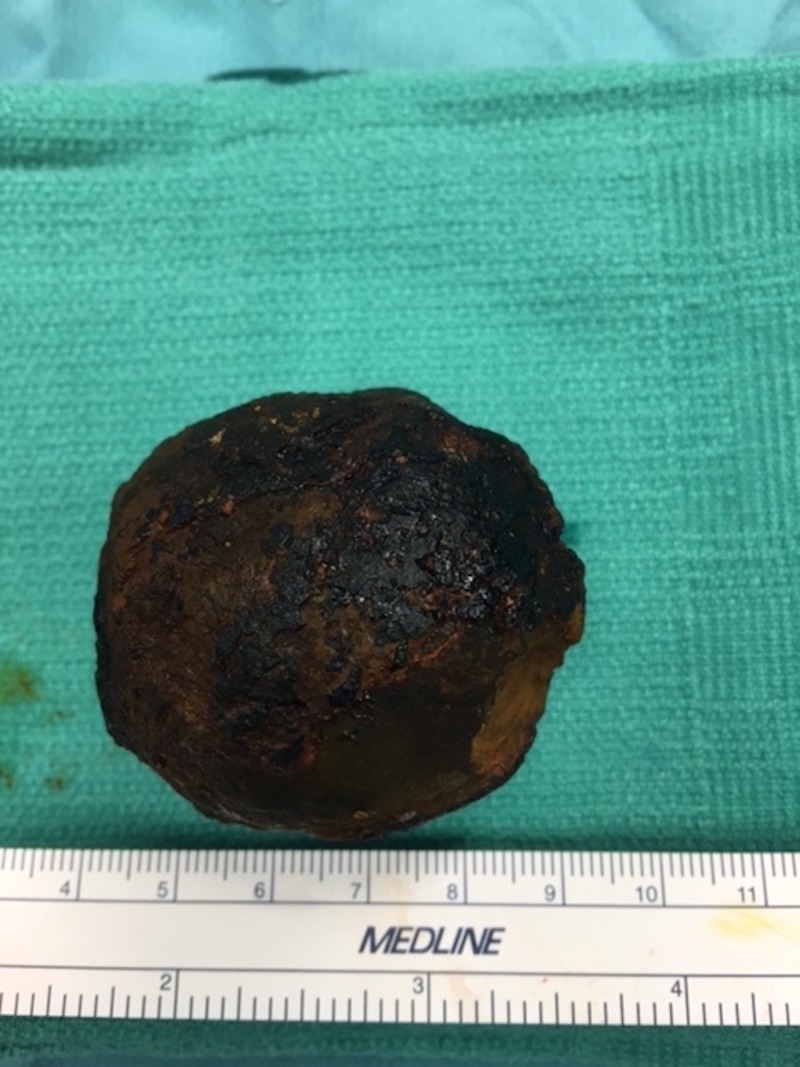
Stone retrieved via surgical duodenotomy

Diagnostic laparoscopy was attempted but failed due to the difficulty accessing the area of interest laparoscopically. Thus, an exploratory laparotomy was carried out. A midline incision was made and dissection was carried down until the duodenum was once again palpated. Attempts were made trying to move the stone proximally into the stomach; however, there was significant inflammation, as well as adhesions, in the duodenum from the longstanding cholecystitis and cholecystoduodenal fistula. Multiple attempts were made to try to lyse the adhesions, trying to free the angle into the stomach or make it less acute. Despite lysing all the adhesions and performing a limited Kocher maneuver, the attempts to move the stone into the stomach were unsuccessful. The surgical team then attempted to pass the stone more distally in an attempt to take it out through a jejunal enterotomy. The stoned moved freely down to D3/D4, but unfortunately, we were unable to move the stone beyond the ligament of Treitz. The ligament of Treitz was taken down, along with adhesions in the area; however, we were still unable to remove the stone. As there was some hematoma forming in this area, we elected to then proceed with a duodenotomy to retrieve the stone. The stone was moved back to D2, and electrocautery was used to make a 5 cm duodenotomy on the lateral antimesenteric border of the duodenum. The stone was encountered, freed, and extracted from the duodenum (Figure 4). Repair of the duodenum and omental flap creation were performed. This was followed by placement of a gastrojejunostomy feeding tube.

## Discussion

Bouveret syndrome is named after Leon Bouveret who published two case studies of this condition in 1896 [6]. Bouveret syndrome is a gastric outlet obstruction caused by gallstone erosion through the intestinal wall through bilioenteric fistula [2, 4]. Chronic cholodocolithiasis causes chronic inflammation, ischemia, and necrosis leading to erosion of the walls of the gallbladder and the duodenum [2]. Gallstones are more common in women resulting in a higher frequency of Bouveret syndrome. A recent review of all published reports of 128 Bouveret cases since 1974 indicated that it is more common in the elderly with a mean age of 74.1 years (standard deviation (SD) = 11) [5]. Bouveret syndrome usually presents similarly to bowel obstruction with symptoms, including nausea, non-bilious vomit, dehydration secondary to vomiting, and epigastric abdominal pain. Symptoms are usually intermittent due to variation in the degree of inflammation and edema surrounding the stone; thus, diagnosis can be challenging. Abdominal radiography will usually show pneumobilia, ectopic gallstone, and gallstone ileus, which is known as Rigler’s triad [2-3]. However, only one-fifth of the cases are diagnosed via abdominal x-ray. Most cases require extensive workup that include CT of the abdomen, right upper quadrant ultrasound, and esophagogastroduodenoscopy [3]. Endoscopy can be diagnostic and therapeutic, but studies have shown that only 10% are successfully retrieved via endoscopic approach and 90% of the cases required surgical intervention. The most common surgical approach includes milking the stone back to the stomach and performing a gastrotomy or moving the stone to the jejunum and performing jejunal enterolithotomy. Usually, duodenotomy is not the preferred approach due to the retroperitoneal orientation of D2 - D4. Cholecystectomy and fistula closure are not usually required [1, 3]. Bouveret syndrome has a mortality of around 12% usually due to the patient's advanced age and co-morbidities. Our case is unique because of the surgical approach that took place to extract the stone (Figure 4). Hence, instead of performing a gastrotomy or jejunal enterotomy, the stone was extracted via duodenotomy at the level of D2 for the reasons we described above.

## Conclusions

Bouveret is one of the rare causes of small bowel obstruction. One should be more vigilant with elderly patients with small bowel obstruction and to include Bouveret or gallstone ileus in the differential diagnosis. Patients should be encouraged to participate in the definitive treatment as soon as possible and to avoid any delay. A delay in the treatment will likely lead to complicated and life-threatening operations in high-risk populations.
